# A fractional order mathematical model for COVID-19 dynamics with quarantine, isolation, and environmental viral load

**DOI:** 10.1186/s13662-021-03265-4

**Published:** 2021-02-11

**Authors:** Mohammed A. Aba Oud, Aatif Ali, Hussam Alrabaiah, Saif Ullah, Muhammad Altaf Khan, Saeed Islam

**Affiliations:** 1 Department of Mathematics and Statistics, Al Imam Mohammad Ibn Saud Islamic University (IMSIU), Riyadh, Kingdom of Saudi Arabia; 2 Department of Mathematics, Abdul Wali Khan University Mardan, Khyber Pakhtunkhwa, 23100 Mardan, Pakistan; 3 College of Engineering, Al Ain University, Al Ain, United Arab Emirates; 4 Mathematics Department, Tafila Technical University, Tafila, Jordan; 5 Department of Mathematics, University of Peshawar, Peshawar, 25000 Pakistan; 6 Informetrics Research Group, Ton Duc Thang University, Ho Chi Minh City, Vietnam; 7 Faculty of Mathematics and Statistics, Ton Duc Thang University, Ho Chi Minh City, Vietnam

**Keywords:** Caputo fractional model, COVID-19, Stability analysis, Real data, Quarantine and isolation, Environmental impact, Parameter estimations, Simulation

## Abstract

COVID-19 or coronavirus is a newly emerged infectious disease that started in Wuhan, China, in December 2019 and spread worldwide very quickly. Although the recovery rate is greater than the death rate, the COVID-19 infection is becoming very harmful for the human community and causing financial loses to their economy. No proper vaccine for this infection has been introduced in the market in order to treat the infected people. Various approaches have been implemented recently to study the dynamics of this novel infection. Mathematical models are one of the effective tools in this regard to understand the transmission patterns of COVID-19. In the present paper, we formulate a fractional epidemic model in the Caputo sense with the consideration of quarantine, isolation, and environmental impacts to examine the dynamics of the COVID-19 outbreak. The fractional models are quite useful for understanding better the disease epidemics as well as capture the memory and nonlocality effects. First, we construct the model in ordinary differential equations and further consider the Caputo operator to formulate its fractional derivative. We present some of the necessary mathematical analysis for the fractional model. Furthermore, the model is fitted to the reported cases in Pakistan, one of the epicenters of COVID-19 in Asia. The estimated value of the important threshold parameter of the model, known as the basic reproduction number, is evaluated theoretically and numerically. Based on the real fitted parameters, we obtained $\mathcal{R}_{0} \approx 1.50$. Finally, an efficient numerical scheme of Adams–Moulton type is used in order to simulate the fractional model. The impact of some of the key model parameters on the disease dynamics and its elimination are shown graphically for various values of noninteger order of the Caputo derivative. We conclude that the use of fractional epidemic model provides a better understanding and biologically more insights about the disease dynamics.

## Introduction

The novel virus (2019-nCoV) that is highly transmissible and pathogenic was first identified from a single individual in Wuhan city in China. This novel infection causes a severe acute respiratory syndrome and it has spread across the world. The reported COVID-19 confirmed cases are over 10.27 million, and there have been more than 0.5 million deaths till 30 June 2020 globally so far [1]. The worst affected regions due to coronavirus are America, Europe, Africa, South-East Asia, Western Pacific, Eastern, and Mediterranean. The initial symptoms of a COVID-19 infection include dry cough, fever, fatigue, and breath shortening that appear in 2–10 days and further cause pneumonia, SARS, kidney failure, and even death [2]. The pandemic has continuously spread due to absence of vaccine and antiviral treatments. Thus WHO announced it a global issue. The policy makers have implemented the non-pharmaceutical intervention like social distancing, self-quarantine, isolation of infected, wearing mask, protective kits for medical personnel, and travel restrictions to minimize the disease incidence. It is also a challenging problem for scientists and virologist evaluating potential treatments based on ongoing clinical trials.

Researchers suggested many mathematical models to analyze the dynamical behavior and spread of the novel virus which can help to predict the future situation and even control of the COVID-19 pandemic [3]. In the analysis of mathematical models of coronavirus, the reproductive number has a significant role in describing the nonlinear dynamics of physical and biological engineering problems. The reproduction number indicates that COVID-19 has been continuously increasing or has been controlled. In Pakistan, 209,337 confirmed infected cases have been reported and about 4304 have lost their lives out of over 220 million population to date [4, 5]. The first case was reported in Karachi on 26 February 2020, and day by day situation is getting worse and virus is spreading quickly due to limiting testing. The government is unable to maintain strict lockdown and has imposed a smart lockdown by easing restrictions due to severe economic hardships, especially for labor community who earns for living to survive every day. To study the dynamics of COVID-19 transmission pattern, many mathematical models provide more insight on how to control the disease spread to health authorities [6–8]. Fanelli and Piazza [9] studied a novel compartmental model describing the transmission patterns of COVID-19 in three highly infected countries. The dynamics of COVID-19 with an impact of non-pharmaceutical interventions was studied by Ullah and Khan [10] on Pakistani data. The fractional mathematical models rendering the natural fact in a systematic way as in [11, 12] and [13, 14] are used to simulate the transmission of coronavirus. Different mathematical models with an effect of nonlocality and fading memory process by using differential operators have been presented [14, 15]. The fractional order epidemic models are more helpful and reliable in analyzing the dynamics of an infectious disease than the classical integer order models [16, 17]. The fractional order models for different diseases show cooperatively better fit to the real data. In [18, 19] a different fractional operator is suggested, and applications of these fractional operators are found in [20, 21]. Recently a Caputo fractional order COVID-19 model has been studied in [22]. Some other fractional mathematical models for the investigations of infectious diseases have been studied in [15, 23–26]. For example, the fractional diffusion equations and their analysis are studied in [15]. A new scheme to solve numerically the fractional order differential equations is utilized in [23]. New numerical investigations for the fluid in non-conventional media are suggested in [24]. Coronavirus modelings, simulations, and their possible control through a mathematical model are studied by the author in [25]. The spread of coronavirus in South Africa and Turkey with detailed statistical and mathematical results is studied in [26]. Recently, the authors have studied the analysis of coronavirus model in fractional derivative [27]. The use of quarantine and isolations in the modeling of coronavirus is investigated in [28]. A mathematical model for the dynamical analysis of coronavirus and its control analysis is studied by the authors in [10]. The notified cases of coronavirus in Saudi Arabia through a mathematical model are considered in [29], where the authors provide suggestions on possible controls based on the parameters.

Environmental viral load plays an essential role in the disease incidence and is considered to be one of the main transmission routes of COVID-19. In this study, we reformulate the model [28] with the impact of quarantine, isolation, and environmental effects on the transmission dynamics of coronavirus with the application of Caputo derivative. The parameter values are estimated from the cumulative COVID-19 cases reported in Pakistan. The fractional order models provide better understanding and give more insights about the pandemic. The rest of the work is arranged as follows: In Sect. 2 basics preliminaries are presented, while the model formulation for integer case with parameter estimation and curve fitting is presented in Sect. 3. Model derivation and basics properties are presented in Sects. 4 and 5, respectively. In Sect. 6 we present the analysis of the model, while the numerical simulations are depicted in Sect. 7. Brief concluding remarks are presented in Sect. 8.

## Preliminaries on fractional derivative

In order to proceed, first we recall some basic definitions regarding fractional calculus.

### Definition 1

The fractional order derivative in the Caputo case with order *α* for a function $g\in C^{n}$ is defined as follows [19]:

1 $$\begin{aligned} {}^{C}D^{\alpha }_{t} \bigl(g(t) \bigr)= \frac{1}{\Gamma (n-\alpha )} \int _{0}^{t} \frac{g^{n}(\varsigma ) (t-\varsigma )^{n-\alpha }}{(t-\varsigma )}\,d \varsigma,\quad n-1< \alpha \leq n\in N. \end{aligned}$$

Clearly, ${}^{C}D^{\alpha }_{t}(g(t))$ tends to $g'(t)$ as $\alpha \rightarrow 1$.

### Definition 2

The corresponding integral with order $\alpha >0$ is defined as follows:

2 $$\begin{aligned} I^{\alpha }_{t} \bigl(g(t) \bigr)= \frac{1}{\Gamma (\alpha )} \int _{0}^{t} \frac{g(\varsigma )(t-\varsigma )^{\alpha }}{(t-\varsigma )}\,d \varsigma, \quad 0< \alpha < 1, t>0. \end{aligned}$$

### Definition 3

The Atangana–Baleanu–Caputo (ABC) fractional operator of order $\alpha \in [0,1]$ is defined as follows [18]:

3 $$\begin{aligned} {}^{{ABC}}_{a}D^{\alpha }_{t} \bigl(g(t) \bigr)= \frac{ABC(\alpha )}{(1-\alpha )} \int _{a}^{t} g^{\prime }(\varsigma ){E}_{ \alpha } \biggl[-\alpha \frac{(t-\varsigma )^{\alpha }}{1-\alpha } \biggr]\,d \varsigma. \end{aligned}$$

### Definition 4

The associated ABC fractional integral of order *α* is defined as follows:

4 $$\begin{aligned} {}^{{ABC}}_{a}I^{\alpha }_{t} \bigl(g(t) \bigr)= \frac{1-\alpha }{B(\alpha )} {g(t)}+ \frac{\alpha }{B(\alpha ){\Gamma (\alpha )}} \int _{a}^{t} g( \varsigma ){(t-\varsigma )^{\alpha -1}}\,d \varsigma,\quad \alpha \in [0,1]. \end{aligned}$$

### Definition 5

The constant point $z^{*}$ is an equilibrium point of the Caputo-fractional model, then [30]

5 $$\begin{aligned} {}^{C}D^{\alpha }_{t}z(t)=g \bigl(t,z(t) \bigr), \quad \alpha \in (0,1), \end{aligned}$$

if and only if $g(t,z^{*})=0$.

## The classical integer order model formulation

In this section, we briefly discuss the integer order model of the dynamics of COVID-19 with quarantine, isolation, and environmental load which is mainly studied for the Chinese population data in the case of fractal-fractional Atangana–Baleanu derivative [28]. For the mathematical model formulation, the net population $N(t)$ at time *t* is further divided into mutually-exclusive sub-compartments as susceptible or healthy $S(t)$, exposed $E(t)$, infected with clinical symptoms $I(t)$, asymptomatically infected $A(t)$, quarantined class $Q(t)$, hospitalized $H(t)$, and the recovered $R(t)$ individuals. The class $M (t)$ denotes the environmental viral load due to the infected people. It is to be that the asymptomatic infected individuals are also capable of transmitting the infection. The nonlinear ordinary differential equations governed by these assumptions are described as follows:

6 $$\begin{aligned} \textstyle\begin{cases} \frac{dS}{dt} = \Lambda - \delta S(t)-\lambda S(t), \\ \frac{dE}{dt} = \lambda S(t) - ((1-\varphi )\omega + \varphi \rho + \delta +\varepsilon _{1}) E(t), \\ \frac{dI}{dt} = (1-\varphi )\omega E(t) -(\sigma _{1}+\delta + \varsigma _{1}+\tau ) I(t), \\ \frac{dA}{dt} = \varphi \rho E(t) - (\sigma _{2}+\delta )A(t), \\ \frac{dQ}{dt} = \varepsilon _{1} E(t) - (\delta +\eta _{1}+ \varepsilon _{2})Q(t), \\ \frac{dH}{dt} = \tau I(t)+\varepsilon _{2} Q(t)- ( \delta +\eta _{2}+ \varsigma _{2})H(t), \\ \frac{dR}{dt} = \sigma _{1} I(t)+\sigma _{2} A(t)+\eta _{1} Q(t)+ \eta _{2} H(t)-\delta R(t), \\ \frac{dM}{dt} = m_{1} I + m_{2} A(t)-m_{3} M(t), \end{cases}\displaystyle \end{aligned}$$

where

7 $$\begin{aligned} \lambda =\frac{\zeta _{1}(I+\psi A)}{N}+\zeta _{2} M, \end{aligned}$$

subject to the following nonnegative initial conditions:

8 $$\begin{aligned} \textstyle\begin{cases} S(0)={S}_{0}\geq 0,\qquad E(0)={E}_{0}\geq 0,\qquad I(0)={I}_{0}\geq 0,\\ A(0)={A}_{0} \geq 0, \qquad Q(0)={Q}_{0}\geq 0, \\ H(0)={H}_{0}\geq 0,\qquad R(0)={R}_{0}\geq 0,\qquad M(0)={M}_{0}\geq 0. \end{cases}\displaystyle \end{aligned}$$

The biological description of the parameters involved in COVID-19 model (6) is given in Table 1.

**Table 1:** Description of the model parameters and their estimated and fitted values

Parameter	Description	Value (in days)	Reference
Λ	Recruitment rate	*δ* × *N*(0)	Estimated
*δ*	Natural death rate	1/(67.7 × 365)	[4]
*ω*	Incubation period	0.1897	[4]
*ρ*	Incubation period	0.1305	Fitted
$\sigma _{1}$	Infected recovery rate	0.5253	Fitted
$\sigma _{2}$	Asymptomatic recovery rate	0.3851	Fitted
$\eta _{1}$	Quarantined recovery rate	0.6715	Fitted
$\eta _{2}$	Recovery rate of the hospitalized	0.6052	Fitted
*τ*	Rate of moving from *I* to *H* class	0.3061	Fitted
$\varepsilon _{1}$	Quarantine rate of exposed individuals	0.2395	Fitted
$\varepsilon _{2}$	Hospitalization rate of *Q* individuals	0.3101	Fitted
$\varsigma _{1}$	Infected disease death rate	0.0222	Fitted
$\varsigma _{2}$	Hospitalized disease death rate	0.0661	Fitted
$\zeta _{1}$	Contact rate	0.6104	Fitted
$\zeta _{2}$	Disease transmission coefficient	1.2 × 10^−7^	Fitted
$m_{1}$	Viral contribution to *M* by *I* class	0.2157	Fitted
$m_{2}$	Viral contribution to *M* by *A* class	0.2276	Fitted
$m_{3}$	Removal rate of virus from *M*	0.2276	Fitted
*ψ*	Transmissibility multiple	0.5856	Fitted

### Parameter estimation

**Figure 1:**
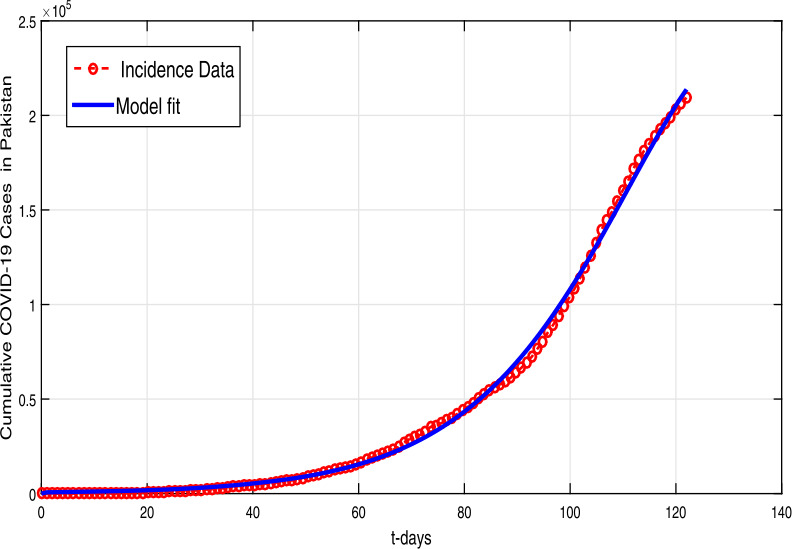
Curve fitting (solid red line) to the confirmed infected cases using model (6), while the data are consider from 1 March to 30 June 2020

In this section, we estimate the model parameters with the help of the well-known statistical technique known as nonlinear least square curve fitting approach. The confirmed infected cases from 1 March to 30 June 2020 in Pakistan are used to estimate the model parameter values [31]. The recruitment rate and the natural mortality rate are estimated from literature, and other parameters are fitted from real data. The updated reproduction number evaluated by using the estimated and fitted parameters is $\mathcal{R}_{0}\approx 1.50$. The predicted curve having a better agreement to the actual reported cases is depicted in Fig. 1. The model parameter values are shown in Table 1. The initial conditions used in data fitting as well as in numerical results are given as $S(0)=220\text{,}870\text{,}336, E(0)=20\text{,}000, I(0)=4, A(0)=200, Q(0)=H(0)=M(0)=0$.

## Model derivation in Caputo operator

We reformulate the COVID-19 fractional order (6) model using a Caputo fractional derivative in order to observe the memory effects and gain more insights about the pandemic. System (6) in terms of integral form is followed by substituting the value of kernel as a power-law correlation function. After applying the Caputo fractional derivative of order $\alpha -1$, we get

9 $$\begin{aligned} \textstyle\begin{cases} {}^{C}D^{\alpha -1}_{t} [\frac{dS}{dt} ] = {}^{C}D^{\alpha -1}_{t}I^{-( \alpha -1)}_{t} [{\Lambda - \delta S(t)-\lambda S(t) } ], \\ {}^{C}D^{\alpha -1}_{t} [\frac{dE}{dt} ] ={}^{C}D^{\alpha -1}_{t}I^{-( \alpha -1)}_{t} [\lambda S(t) - ((1-\varphi )\omega + \varphi \rho +\delta +\varepsilon _{1}) E(t) ], \\ {}^{C}D^{\alpha -1}_{t} [\frac{dI}{dt} ] = {}^{C}D^{\alpha -1}_{t}I^{-( \alpha -1)}_{t} [{(1-\varphi )\omega E(t) -(\sigma _{1}+\delta + \varsigma _{1}+\tau ) I(t)} ], \\ {}^{C}D^{\alpha -1}_{t} [\frac{dA}{dt} ] ={}^{C}D^{\alpha -1}_{t}I^{-( \alpha -1)}_{t} [\varphi \rho E(t) - (\sigma _{2}+\delta )A(t) ], \\ {}^{C}D^{\alpha -1}_{t} [\frac{dQ}{dt} ] = {}^{C}D^{\alpha -1}_{t}I^{-( \alpha -1)}_{t} [\varepsilon _{1} E(t) - (\delta +\eta _{1}+ \varepsilon _{2})Q(t) ], \\ {}^{C}D^{\alpha -1}_{t} [\frac{dH}{dt} ] ={}^{C}D^{\alpha -1}_{t}I^{-( \alpha -1)}_{t} [\tau I(t)+\varepsilon _{2} Q(t)- ( \delta +\eta _{2}+ \varsigma _{2})H(t) ], \\ {}^{C}D^{\alpha -1}_{t} [\frac{dR}{dt} ] ={}^{C}D^{\alpha -1}_{t}I^{-( \alpha -1)}_{t} [\sigma _{1} I(t)+\sigma _{2} A(t)+\eta _{1} Q(t)+ \eta _{2} H(t)-\delta R(t) ], \\ {}^{C}D^{\alpha -1}_{t} [\frac{dM}{dt} ] ={}^{C}D^{\alpha -1}_{t}I^{-( \alpha -1)}_{t} [m_{1} I + m_{2} A(t)-m_{3} M(t) ]. \end{cases}\displaystyle \end{aligned}$$

Both are the inverse operators, we get the COVID-19 model in the Caputo operator form

10 $$\begin{aligned} \textstyle\begin{cases} {}^{C}D^{\alpha }_{t}S(t)={\Lambda - \delta S(t)-\lambda S(t) }, \\ {}^{C}D_{t}^{\alpha }E(t)=\lambda S(t) - ((1-\varphi )\omega + \varphi \rho +\delta +\varepsilon _{1}) E(t), \\ {}^{C}D_{t}^{\alpha }I(t)=(1-\varphi )\omega E(t) -(\sigma _{1}+\delta + \varsigma _{1}+\tau ) I(t), \\ {}^{C}D_{t}^{\alpha }A(t)=\varphi \rho E(t) - (\sigma _{2}+\delta )A(t), \\ {}^{C}D_{t}^{\alpha }Q(t)=\varepsilon _{1} E(t) - (\delta +\eta _{1}+ \varepsilon _{2})Q(t), \\ {}^{C}D_{t}^{\alpha }H(t)=\tau I(t)+\varepsilon _{2} Q(t)- ( \delta + \eta _{2}+\varsigma _{2})H(t), \\ {}^{C}D_{t}^{\alpha }R(t)=\sigma _{1} I(t)+\sigma _{2} A(t)+\eta _{1} Q(t)+ \eta _{2} H(t)-\delta R(t), \\ {}^{C}D_{t}^{\alpha }M(t)=m_{1} I + m_{2} A(t)-m_{3} M(t). \end{cases}\displaystyle \end{aligned}$$

The initial conditions are as follows:

11 $$\begin{aligned} \begin{aligned} &S(0) \geq 0, \qquad E(0) \geq 0,\qquad I(0) \geq 0,\qquad A(0) \geq 0, \\ &Q(0) \geq 0,\qquad H(0) \geq 0,\qquad R(0) \geq 0,\qquad M (0)\geq 0. \end{aligned} \end{aligned}$$

## Basic properties

### Invariant region

The dynamics of Caputo fractional model (10) is explored in a feasible region $\Omega \subset \mathbb{R}_{+}^{8}$ such that $\Omega = \{(S(t),E(t),I(t),A(t),Q(t),H(t),R(t))\in \mathbb{R}_{+}^{7}:N(t) \leq \frac{\Lambda }{\delta }, M(t)\in \mathbb{R}_{+}:\leq \frac{\Lambda }{\delta }\frac{m_{1}+m_{2}}{m_{3}} \}$.

#### Lemma 1

*The region* $\Omega \subset \mathbb{R}_{+}^{7}$ *is positively invariant with nonnegative initial conditions for model* (10) *in* $\mathbb{R}_{+}^{7}$.

#### Proof

After summing the components of human population in model (10), we obtain a total population as follows:

$$\begin{aligned} {}^{C}D_{t}^{\alpha }N(t)={}&{}^{C}D_{t}^{\alpha }S(t)+{}^{C}D_{t}^{\alpha }E(t)+{}^{C}D_{t}^{\alpha }I(t)+{}^{C}D_{t}^{\alpha }A(t)+{}^{C}D_{t}^{\alpha }Q(t) \\ &{}+{}^{C}D_{t}^{\alpha }H(t)+{}^{C}D_{t}^{\alpha }R(t), \end{aligned}$$

and then we have

$$\begin{aligned} {}^{C}D_{t}^{\alpha }N(t)+\delta N(t)\leq \Lambda. \end{aligned}$$

After applying the Laplace transform, we have

$$\begin{aligned} N(s)\leq \frac{\Lambda }{s(s^{\alpha }+\delta )}+N(0){ \frac{s^{\alpha -1}}{s^{\alpha } + \delta }}. \end{aligned}$$

By considering the inverse Laplace, we arrive at

$$\begin{aligned} N(t)\leq N(0) E_{\alpha,1} \bigl(\delta t^{\alpha } \bigr)+\Lambda t^{\alpha }E_{ \alpha,\alpha +1} \bigl(\delta t^{\alpha } \bigr), \end{aligned}$$

where the Mittag-Leffler function is shown by

$$\begin{aligned} E_{\alpha,\beta (y)}=\sum^{\infty }_{n=0} { \frac{y^{n}}{\Gamma (\alpha n+\beta )}}, \end{aligned}$$

and the Laplace transform is

$$\begin{aligned} L \bigl[t^{{\beta } -1}{E_{{\alpha },\beta } \bigl(\pm \alpha t^{\alpha } \bigr)} \bigr]= \frac{s^{{\alpha }-{\beta }}}{s^{\alpha }\mp \alpha }. \end{aligned}$$

Thus, the solution of the model with the nonnegative conditions in Ω remains in Ω. So, the region Ω is positively invariant and attracts all the solutions in $\mathbb{R}_{+}^{7}$. □

Now, for the positivity of the system solution, let

$$\begin{aligned} \mathbb{R}^{7}_{+}= \bigl\{ y\in \mathbb{R}^{7} \mid y \geq 0 \bigr\} \quad\text{and}\quad y(t)= \bigl(S(t),E(t),I(t),A(t),Q(t),H(t),R(t) \bigr)^{T}. \end{aligned}$$

#### Corollary 1

[32] *Suppose that* $g(t) \in C[m, n]$ *and* ${}^{C}D_{t}^{\alpha }g(t) \in (m, n]$,*where* $\alpha \in (0, 1]$. *Then if*

$$\begin{aligned} &\mathrm{(i)}.\quad {}^{C}D_{t}^{\alpha }g(t)\geq 0,\quad \forall y\in (m,n),\textit{then }g(t)\textit{ is nondecreasing}; \\ &\mathrm{(ii)}.\quad {}^{C}D_{t}^{\alpha }g(t)\leq 0,\quad \forall y\in (m,n),\textit{then }g(t)\textit{ is nonincreasing}. \end{aligned}$$

### Positivity and boundedness

#### Proposition 1

*The solution of model* (10) *is nonnegative and bounded for all* $(S(0), E(0), I(0), A(0), Q(0), H(0),R(0)) \in \mathbb{R}_{+}^{7}$ *for* $t>0$.

#### Proof

To show that the solution of the model is nonnegative, it is required to show that on each hyper-plane bounding the positive orthant there is a the vector field point $\mathbb{R}^{7}_{+}$. From system (10), we have

$$\begin{aligned} &{}^{C}D_{t}^{\alpha }S\mid _{S=0}=\Lambda > 0,\qquad {}^{C}D_{t}^{\alpha }E \mid _{E=0}= \lambda S\geq 0,\qquad {}^{C}D_{t}^{\alpha }I\mid _{I=0}=(1- \varphi )\omega E\geq 0, \\ &{}^{C}D_{t}^{\alpha }A\mid _{A=0}=\varphi \rho E \geq 0,\qquad {}^{C}D_{t}^{\alpha }Q\mid _{Q=0}=\varepsilon _{1} E\geq 0,\qquad {}^{C}D_{t}^{\alpha }H \mid _{H=0}=\tau I+ \varepsilon _{2} Q \geq 0, \\ &{}^{C}D_{t}^{\alpha }R\mid _{R=0}=\sigma _{1} I+\sigma _{2} A+ \eta _{1} Q + \eta _{2} H\geq 0. \end{aligned}$$

The solution is

$$\begin{aligned} N(t)\leq N(0) E_{\alpha,1} \bigl(-\delta t^{\alpha } \bigr)+\Lambda t^{\alpha }E_{ \alpha,\alpha +1} \bigl(-\delta t^{\alpha } \bigr). \end{aligned}$$

Also, the Mittag-Leffler function is bounded ∀ $t>0$. Therefore, we have

$$\begin{aligned} \lim_{t\rightarrow +\infty } N(t)\leq \frac{\Lambda }{\delta }. \end{aligned}$$

The solution of the system will remain in $R^{7}_{+}$ by using Corollary 1, and hence a biologically feasible region is constructed as follows:

$$\begin{aligned} \Omega = \bigl\{ (S,E,I,A,Q,H,R )\in \mathbb{R}^{7}_{+}:S,E,I,A,Q,H,R \geq 0 \bigr\} . \end{aligned}$$

Since all the terms are positive, the solution of model (10) is bounded. □

## Analysis of the model

### Disease-free equilibrium (DFE)

For the equilibrium points of fractional model (10), we have

$$\begin{aligned} {}^{C}D^{\alpha }_{t}S(t)={}&{}^{C}D^{\alpha }_{t}E(t)={}^{C}D^{\alpha }_{t}I(t)= {}^{C}D^{\alpha }_{t}A(t)={}^{C}D^{\alpha }_{t}Q(t)= {}^{C}D^{\alpha }_{t}H(t)={}^{C}D^{ \alpha }_{t}R(t) \\ ={}&{}^{C}D^{\alpha }_{t}M(t)=0. \end{aligned}$$

The disease-free equilibrium is denoted by $E_{0}^{*}=(S^{0},E^{0},I^{0},A^{0},Q^{0},H^{0},R^{0},M^{0})$ and is given by

12 $$\begin{aligned} E_{0}^{*}= \biggl({ \frac{\Lambda }{\delta },0,0,0,0,0,0,0} \biggr). \end{aligned}$$

### The reproduction number

The next generation technique is used to derive the reproduction number $\mathcal{R}_{0}$ for the dynamics of disease. Let $x=(E,I,A,Q,H,M)^{T}$, then we have

13 $$\begin{aligned} \frac{dx}{dt}=\mathbf {F}-\mathbf {V}, \end{aligned}$$

where

$$\begin{aligned} &\mathbf{F}= \begin{pmatrix} 0 & \zeta _{1} & \psi \zeta _{1} & 0 & \frac{\Lambda \zeta _{2}}{\delta } & 0 \\ 0 & 0 & 0 & 0 & 0 & 0 \\ 0 & 0 & 0 & 0 & 0 & 0 \\ 0 & 0 & 0 & 0 & 0 & 0 \\ 0 & 0 & 0 & 0 & 0 & 0 \\ 0 & 0 & 0 & 0 & 0 & 0 \end{pmatrix}, \\ &\mathbf{V} = \begin{pmatrix} k_{0} & 0 & 0 & 0 & 0 & 0 \\ (\varphi -1)\omega & k_{1} & 0 & 0 & 0 & 0 \\ -\varphi \rho & 0 & k_{2} & 0 & 0 & 0 \\ -\delta _{1} & 0 & 0 & k_{3} & 0 & 0 \\ 0 & -\tau & 0 & -\varepsilon _{2} & k_{4} & 0 \\ 0 & -m_{1} & -m_{2} & 0 & 0 & m_{3} \end{pmatrix}. \end{aligned}$$

The next generation matrix is of the form

$$ \mathbf{F}\mathbf{V}^{-1}= \begin{pmatrix} \frac{\rho \varphi \psi \varsigma _{1}}{k_{0}k_{2}}+ \frac{k_{2}k_{3}k_{4}m_{3}\omega (1-\varphi )\varsigma _{1}}{k_{0}k_{1}k_{2}k_{3}k_{4}m_{3}}+d& \frac{\varsigma _{1}}{k_{1}}+ \frac{m_{1}\Lambda \varsigma _{2}}{k_{1}m_{3}\delta } & \frac{\psi \varsigma _{1}}{k_{2}}+ \frac{m_{2}\Lambda \varsigma _{2}}{k_{2}m_{3}\delta } & 0 & 0 & \frac{\Lambda \zeta _{2}}{m_{3}\delta } \\ 0 & 0 & 0 & 0 & 0 & 0 \\ 0 & 0 & 0 & 0 & 0 & 0 \\ 0 & 0 & 0 & 0 & 0 & 0 \\ 0 & 0 & 0 & 0 & 0 & 0 \\ 0 & 0 & 0 & 0 & 0 & 0 \end{pmatrix}, $$

where

$$\begin{aligned} &k_{0}=(1-\varphi )\omega +\varphi \rho +\delta +\varepsilon _{1},\qquad k_{1}= \sigma _{1}+\delta +\varsigma _{1}+\tau,\qquad k_{2}=\sigma _{2}+\delta, \\ &k_{3}=\delta +\eta _{1}+\varepsilon _{2},\qquad k_{4}=\delta +\eta _{2}+ \varsigma _{2}, \\ &d= \frac{(k_{1}k_{3}k_{4}m_{2}\varphi \rho +k_{2}k_{3}k_{4}m_{1}\omega -k_{2}k_{3}k_{4}m_{1}\omega \varphi )\Lambda \varsigma _{2}}{k_{0}k_{1}k_{2}k_{3}k_{4}m_{3}\delta }. \end{aligned}$$

The spectral radius of the next generation matrix is shown by

14 $$\begin{aligned} \mathcal{R}_{0}={}&\frac{k_{1}\delta m_{3}\zeta _{1}\varphi \rho \psi +k_{2}\delta m_{3}\omega \zeta _{1}-k_{2}\zeta _{2}\varphi \omega \Lambda m_{1} }{k_{0}k_{1}k_{2}m_{3}\delta } \\ &{}+ \frac{k_{1}\zeta _{2}\rho \varphi \Lambda m_{2}+k_{2}\zeta _{2}\omega \Lambda m_{1}-k_{2}\delta \zeta _{1}\varphi m_{3}\omega }{k_{0}k_{1}k_{2}m_{3}\delta }. \end{aligned}$$

### Local stability of DFE

#### Theorem 1

*For any two positive integers* *q* *and* *r*, *let* $gcd(q,r)=1$ *for* $\alpha =\frac{q}{r}$ *and* $M=n$, *the disease*-*free equilibrium is locally asymptotically stable if* $|\arg (\lambda )|>\frac{\pi }{2M}$ *for all roots of the associated characteristic equation*

15 $$\begin{aligned} \det \bigl(\operatorname{diag} \bigl[\lambda ^{M\alpha }\lambda ^{M\alpha }\lambda ^{M\alpha } \lambda ^{M\alpha }\lambda ^{M\alpha }\lambda ^{M\alpha }\lambda ^{M \alpha }\lambda ^{M\alpha } \bigr]-J \bigl(E_{0}^{*} \bigr) \bigr)=0. \end{aligned}$$

#### Proof

For local stability, the linearized system is the Jacobian of system at disease-free state as follows:

16 $$\begin{aligned} J \bigl(E_{0}^{*} \bigr)= \begin{pmatrix} -\delta & 0 & -\zeta _{1} & -\psi \zeta _{1} & 0 & 0 & 0 & \frac{\Lambda \zeta _{2}}{\delta } \\ 0 & -k_{0} & \zeta _{1} & \psi \zeta _{1} & 0 & 0 & 0 & \frac{\Lambda \zeta _{2}}{\delta } \\ 0 & (1-\varphi )\omega & -k_{1} & 0 & 0 & 0 & 0 & 0 \\ 0 & \varphi ) & 0 & -k_{2} & 0 & 0 & 0 & 0 \\ 0 & \varepsilon _{1} & 0 & 0 & -k_{3} & 0 & 0 & 0 \\ 0 & 0 & \tau & 0 & \delta _{2} & -k_{4} & 0 & 0 \\ 0 & 0 & \sigma _{1} & \sigma _{2} & \eta _{1} & \eta _{2} & -\delta & 0 \\ 0 & 0 & m_{1} & m_{2} & 0 & 0 & 0 & -m_{3} \end{pmatrix}. \end{aligned}$$

The evaluation of determinant equation (15) implies

17 $$\begin{aligned} \bigl(\lambda ^{q}+\delta \bigr)^{2} \bigl(\lambda ^{q}+k_{3} \bigr) \bigl( \lambda ^{q}+k_{4} \bigr) \bigl( \lambda ^{4q}+a_{1} \lambda ^{3q}+a_{2}\lambda ^{2q}+ a_{3} \lambda ^{q}+a_{4} \bigr)=0, \end{aligned}$$

the eigenvalues $-\delta,-\delta,-k_{3},-k_{4}$ have negative real parts and the others can be found from the last factor of (17). The co-efficient is of the form

$$\begin{aligned} a_{1} ={}& k_{0} +k_{1}+k_{2}+ m_{3}, \\ a_{2} ={}& k_{0}k_{2}(1-\mathcal{R}_{1})+(k_{0}+k_{1}+k_{2})m_{3}+k_{1}k_{2}, \\ a_{3} ={}&k_{0}k_{1}k_{2}(1- \mathcal{R}_{2})+k_{0}k_{2}m_{3}(1- \mathcal{R}_{3})+k_{0}k_{1}m_{3}(1- \mathcal{R}_{4})+k_{1}k_{2}m_{3} \\ &{}-\zeta _{1} \bigl(\varphi k_{1}\rho \psi +m_{3} \bigl(\varphi \rho \psi +(1- \varphi )\omega \bigr) \bigr), \\ a_{4}={}& k_{0}k_{1}k_{2}m_{3}(1- \mathcal{R}_{0}). \end{aligned}$$

Clearly, $a_{i}$ for $i=1,2,\ldots,4$ are all positive if $\mathcal{R}_{0}<1$. The arguments of the roots of equations $(\lambda ^{q}+\delta )^{2}=0$, $(\lambda ^{q}+k_{3})=0$ and $(\lambda ^{q}+k_{4})=0$ are similar, that is,

$$\begin{aligned} \arg (\lambda _{k})=\frac{\pi }{m}+k\frac{2\pi }{m}> \frac{\pi }{M}> \frac{\pi }{2M},\quad \text{where } k=0,1,2,\ldots,(m-1). \end{aligned}$$

In a similar fashion, we also find that the arguments of the equation $(\lambda ^{4q}+a_{1}\lambda ^{3q}+a_{2}\lambda ^{2q}+a_{3}\lambda ^{q}+a_{4})=0$ are all greater than $\frac{\pi }{2M}$ if $\mathcal{R}_{0} <1$, having an argument less than $\frac{\pi }{2M}$ for $\mathcal{R}_{0} >1$. The DFE is locally asymptotically stable for $\mathcal{R}_{0} <1$. □

### Global stability of DFE

The Lyapunov function approach is used to proceed to the result for the GAS of the proposed model at the disease-free state. For this, we have the following theorem.

#### Theorem 2

*The DFE of the Caputo COVID*-19 *model is GAS if* $\mathcal{R}_{0}< 1$.

#### Proof

Consider the following appropriate Lyapunov function:

18 $$\begin{aligned} \mathfrak{F}(t)=Y_{1}E+Y_{2}I+Y_{3}A+Y_{4}M, \end{aligned}$$

where the coefficients *Yj*, for $j=1, 2, 3, 4$, are unknown positive constants, and they will be chosen later. The Caputo-fractional derivative of $\mathfrak{F}(t)$, along model (10), yields

19 $$\begin{aligned} {}^{C}D^{\alpha }_{t}\mathfrak{F}(t)={}&Y_{1}{}^{C}D^{\alpha }_{t}E+ Y_{2}{}^{C}D^{ \alpha }_{t}I+Y_{3}{}^{C}D^{\alpha }_{t}A+Y_{4}{}^{C}D^{\alpha }_{t}M, \\ {}^{C}D^{\alpha }_{t}\mathfrak{F}(t)={}& Y_{1} \bigl({\lambda S(t)-k_{0}E(t)} \bigr)+Y_{2} \bigl({(1- \varphi )\omega E(t)-k_{1}I(t)} \bigr) \\ &{}+ Y_{3} \bigl({\varphi \rho E(t)-k_{2}A(t)} \bigr) + Y_{4} \bigl({m_{1}I(t)+m_{2}A(t)-m_{3}M(t)} \bigr) \\ \leq{}& (Y_{1}\zeta _{1}-Y_{2}k_{1}+Y_{4}m_{1} )I(t)+ ({Y_{1} \zeta _{1}\psi -Y_{3}k_{2}+Y_{4}m_{2}} )A(t) \\ &{}+ \bigl({Y_{2}(1-\varphi )\omega -Y_{1}k_{0}+Y_{3} \varphi \rho } \bigr)E(t)+ \bigl({\zeta _{2}S^{0}-Y_{4}m_{3}} \bigr)M(t), S \leq N,S^{0} \\ \leq{}& (Y_{1}\zeta _{1}-Y_{2}k_{1}+Y_{4}m_{1} )I(t)+ ({Y_{1} \zeta _{1}\psi -Y_{3}k_{2}+Y_{4}m_{2}} )A(t) \\ &{}+ Y_{1}k_{0} \biggl( \frac{Y_{2}(1-\varphi )\omega +Y_{3}\varphi \rho }{Y_{1}k_{0}}-1 \biggr)E(t)+ \bigl({\zeta _{2}S^{0}-Y_{4}m_{3}} \bigr)M(t). \end{aligned}$$

Let us choose

20 $$\begin{aligned} &Y_{1}=m_{3}\delta,\qquad Y_{2}= \frac{m_{3}\delta \zeta _{1}+\Lambda m_{1}\zeta _{2}}{k_{1}},\qquad Y_{3}={m_{3} \psi \zeta _{1}\delta +\Lambda m_{2}\zeta _{2}} {k_{2}},\qquad Y_{4}= \Lambda \zeta _{2}. \\ & {}^{C}D^{\alpha }_{t}\mathfrak{F}(t)\leq k_{0}m_{3}\delta ( \mathcal{R}_{0}-1 )E. \end{aligned}$$

Hence it follows that ${}^{C}D^{\alpha }_{t}\mathfrak{F}\leq 0$ for $\mathcal{R}_{0}\leq 1$, all the parameters and variables are nonnegative with ${}^{C}D^{\alpha }_{t}\mathfrak{F}=0$ iff $E=I=A=M=0$. Thus $(E,I,A,M)\rightarrow (0,0,0,0)$ as $t\rightarrow \infty $. By using $E=I=A=M=0$ in the first and second last three equations of (10) implies that $S\rightarrow \frac{\Lambda }{\delta }$, and $Q,H,R\rightarrow 0$ as $t\rightarrow \infty $. Thus, using Lyapunov stability theorems for the fractional case developed in [30], the solution of Caputo model (10) with nonnegative initial conditions approaches to $E_{0}^{*}$ as $t\rightarrow \infty $ in a feasible region. Thus, it follows that the DFE of model (10) is GAS. □

### Existence of endemic equilibrium point

We denote the endemic equilibrium for fractional coronavirus model given in (10) by $E^{**}_{01}=(S^{**}, E^{**}, I^{**}, A^{**}, Q^{**},H^{**},R^{**}, M^{**})$ such that

21 $$\begin{aligned} \textstyle\begin{cases} S^{**}=\frac{\Lambda }{\delta +\lambda ^{**}}, \\ E^{**}=\frac{\lambda ^{**}S^{**}}{k_{0}}, \\ I^{**}=\frac{(1-\varphi )\omega E^{**}}{k_{1}}= \frac{(1-\varphi )\omega \lambda ^{**}S^{**}}{k_{0}k_{1}}, \\ A^{**}=\frac{\varphi \rho E^{**} }{k_{2}}=\frac{\varphi \rho \lambda ^{**}S^{**} }{k_{0}k_{2}}, \\ Q^{**}=\frac{\varepsilon _{1} E^{**}}{k_{3}}= \frac{\varepsilon _{1} \lambda ^{**}S^{**}}{k_{0}k_{3}}, \\ H^{**}=\frac{\tau I^{**}+\varepsilon _{2} Q^{**}}{k_{4}}= \frac{\tau k_{3} (1-\varphi )\omega \lambda ^{**}S^{**}+k_{1}\varepsilon _{1}\varepsilon _{2} \lambda ^{**}S^{**}}{k_{0}k_{1}k_{2}k_{3}k_{4}}, \\ M^{**}=\frac{m_{2}A^{**}+m_{1}I^{**}}{m_{3}}= \frac{k_{1}m_{2}\varphi \rho \lambda ^{**}S^{**}+k_{2} m_{1} (1-\varphi )\omega \lambda ^{**}S^{**}}{k_{0}k_{1}k_{2}m_{3}}, \\ R^{**}= \frac{\sigma _{2}A^{**}+\eta _{2} H^{**}+\sigma _{1}I^{**}+\eta _{1}Q^{**}}{\delta }. \end{cases}\displaystyle \end{aligned}$$

Inserting the above result into the force of infection state, we get

22 $$\begin{aligned} \lambda ^{**} =\frac{\zeta _{1}(I^{**}+\psi A^{**}) }{N^{**}}+\zeta _{2}M^{**}. \end{aligned}$$

Substituting (21) into (22) shows that the nonzero equilibria of the model satisfy the quadratic equation

23 $$\begin{aligned} b_{0} {\lambda ^{**}}^{2}+b_{1} \lambda ^{**}+b_{2}=0, \end{aligned}$$

where

$$\begin{aligned} b_{0}={}&k_{0}k_{1}k_{2}m_{3} \bigl(k_{2} \bigl(\varepsilon _{1}k_{1} \bigl( \varepsilon _{2} (\delta +\eta _{2} )+k_{4} ( \delta +\eta _{1} ) \bigr)+k_{3}k_{5} \bigr)+ \varphi k_{1}k_{3}k_{4} \rho (\delta +\zeta _{2} ) \bigr), \\ b_{1}={}&k_{0}k_{1}k_{2}\delta m_{3} \bigl(k_{2} \bigl(\varepsilon _{1}k_{1} \bigl(\varepsilon _{2} (\delta +\eta _{2} )+k_{4} ( \delta +\eta _{1} ) \bigr)+k_{3}k_{7} \bigr)+ \varphi k_{1}k_{3}k_{4} \rho (\delta - \psi \zeta _{1}+\sigma _{2} ) \bigr) \\ &{}+\sigma _{2}k_{6}\Lambda \bigl( (1-\varphi )k_{2}m_{1} \omega -\varphi k_{1}\rho m_{2} \bigr)+{k_{0}^{2}} {k_{1}^{2}} {k_{2}^{2}}k_{3}k_{4} \delta m_{3}, \\ b_{2}={}& {k_{0}^{2}} {k_{1}^{2}} {k_{2}^{2}}k_{3}k_{4}\delta ^{2} m_{3} (1-\mathcal{R}_{0} ). \end{aligned}$$

Also, we have

$$\begin{aligned} &k_{5}=\tau (\delta +\eta _{2}(1-\varphi )\omega +k_{4} \bigl((1-\varphi ) \omega (\delta +\eta _{1})+k_{1} \delta \bigr), \\ &k_{6}=k_{2}(-k_{2} \bigl(\varepsilon _{1}k_{1} \bigl(\varepsilon _{2}(\delta + \eta _{2})+k_{4}(\delta +\eta _{1}) \bigr)-k_{3}k_{5} \bigr)-\varphi k_{1}k_{3}k_{4} \rho (\delta +\sigma _{2}), \\ &k_{7}=\tau (\delta +\eta _{2}(1-\varphi )\omega +k_{4} \bigl((1-\varphi ) \omega (\delta +\eta _{1}-\zeta _{1})+k_{1}\delta \bigr). \end{aligned}$$

Here, it should be noted that the coefficient of (23), $b_{0}>0$, and $b_{2}$ is positive (negative) $\mathcal{R}_{0}<1,(>)$. The following result is established.

#### Theorem 3

*The proposed model has*:

(i) *a unique* $(E_{01}^{**})$ *endemic equilibrium if* $b_{2}<0\Leftrightarrow \mathcal{R}_{0}>1$;
(ii) *unique* $E_{01}^{**}$ *if* $b_{1}<0,\wedge b_{2}=0)\vee b_{1}^{2}-4b_{0}b_{2}=0$;
(iii) *if* $b_{1}<0$, $b_{2}>0$ *and the discriminant is greater than zero*, *then there exist two endemic equilibria*.
(iv) *Otherwise*, *no endemic equilibrium exists*.

Thus from case (i) of Theorem 3, it is clear that model (10) has a unique positive endemic equilibrium when $\mathcal{R}_{0}>1$, and point (iii) indicates the possibility of backward bifurcation when $\mathcal{R}_{0}<1$.

## Numerical results

**Figure 2:**
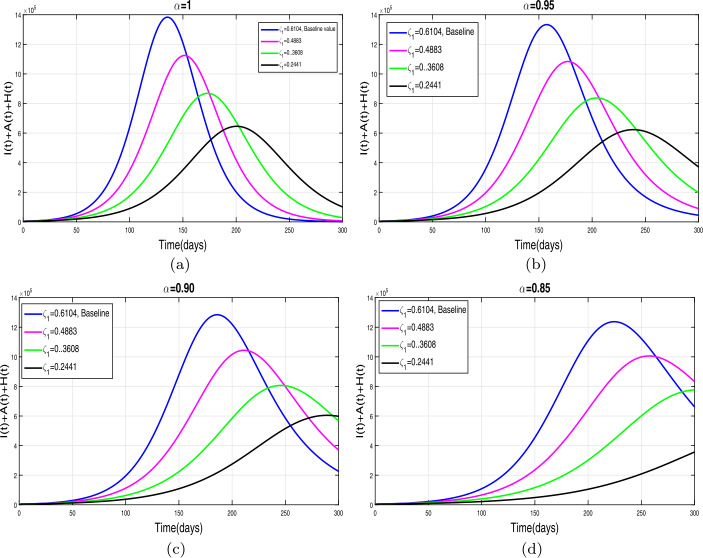
The impact of contact rate $\zeta _{1}$ on cumulative symptomatic, asymptomatic, and hospitalized COVID-19 individuals for $\alpha =1, \alpha =0.95, \alpha =0.90, \alpha =0.85$

**Figure 3:**
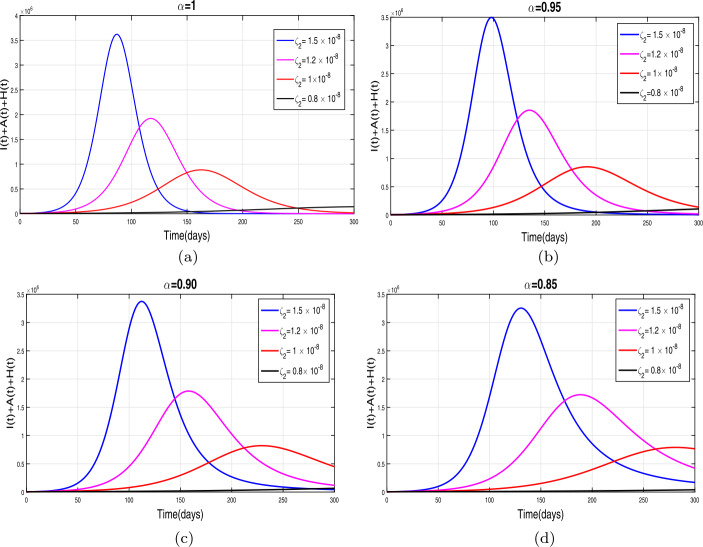
The impact of contact rate $\zeta _{2}$ on cumulative symptomatic, asymptomatic, and hospitalized COVID-19 individuals for $\alpha =1, \alpha =0.95, \alpha =0.90, \alpha =0.85$

**Figure 4:**
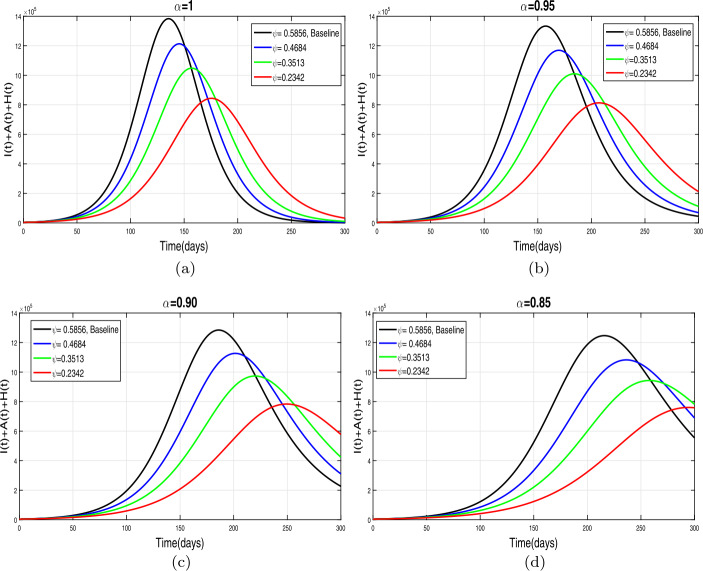
The impact of *ψ* on cumulative symptomatic, *A*, and *H* COVID-19 individuals for $\alpha =1, \alpha =0.95, \alpha =0.90, \alpha =0.85$

**Figure 5:**
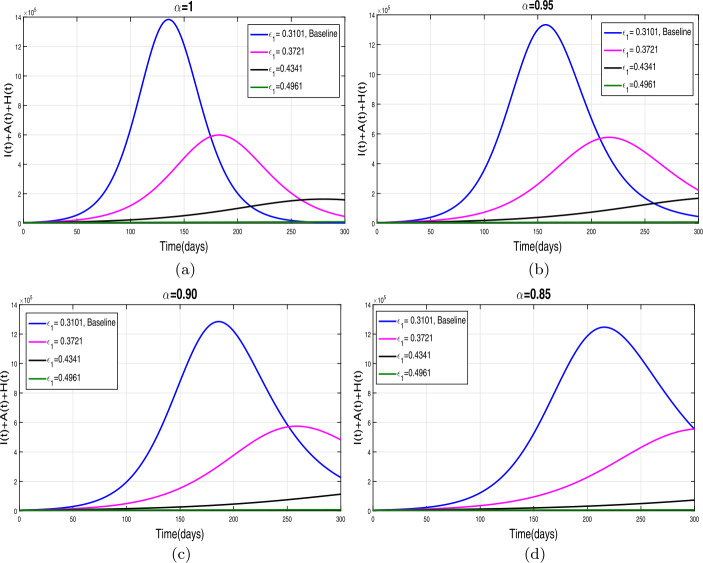
The influence of quarantine rate of exposed individuals $\varepsilon _{1}$ on *I*, *A*, and *H* individuals for $\alpha =1, \alpha =0.95, \alpha =0.90, \alpha =0.85$

**Figure 6:**
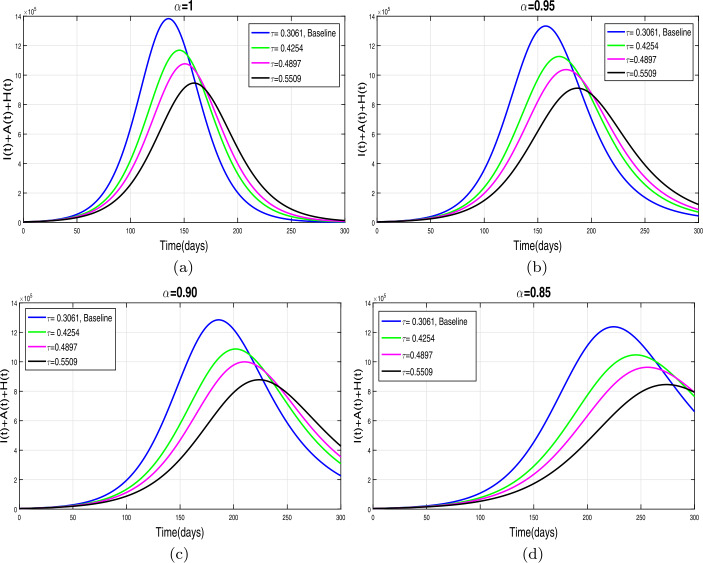
The impact of hospitalization (self-isolation) rate on $I, A$, and *H* individuals for $\alpha =1, \alpha =0.95, \alpha =0.90, \alpha =0.85$

**Figure 7:**
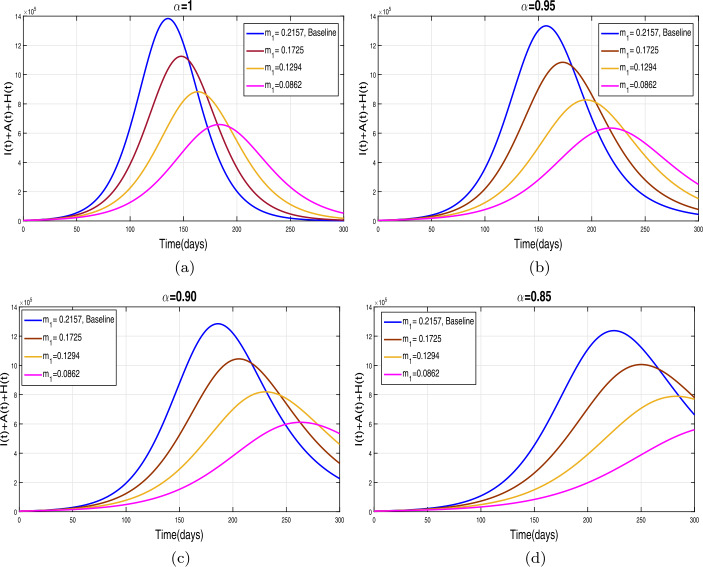
The impact of viral influence of *A* individuals to the environment on $I, A$, and *H* individuals for $\alpha =1, \alpha =0.95, \alpha =0.90, \alpha =0.85$

**Figure 8:**
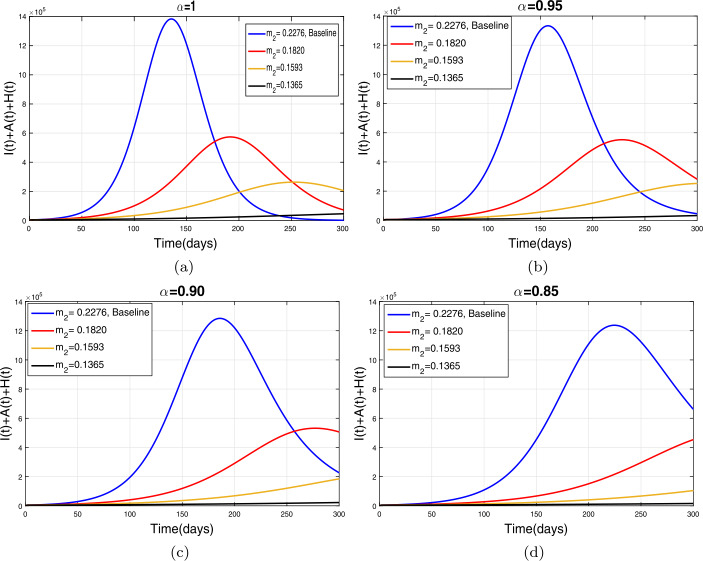
The impact of $m_{2}$ to the environment on symptomatic, asymptomatic, and hospitalized COVID-19 individuals for $\alpha =1, \alpha =0.95, \alpha =0.90, \alpha =0.85$

**Figure 9:**
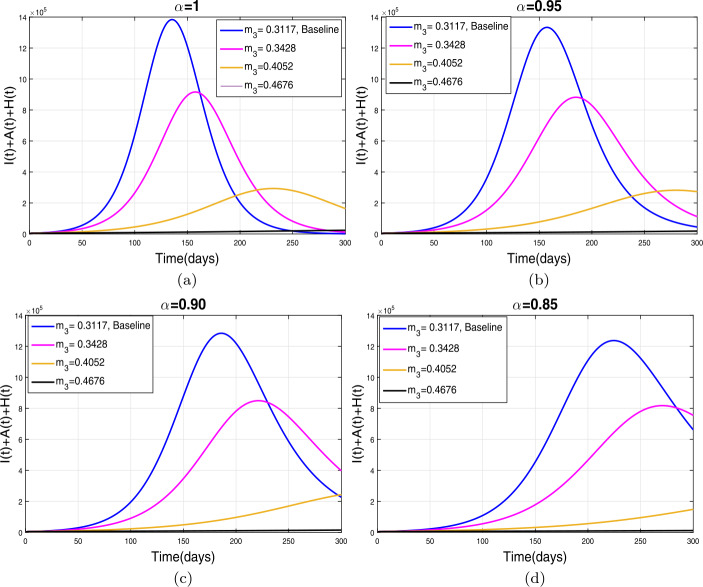
The impact of removal rate $m_{3}$ on symptomatic, *A*, and *H* COVID-19 individuals for $\alpha =1, \alpha =0.95, \alpha =0.90, \alpha =0.85$

This section presents simulation and discussion for the Caputo COVID-19 model (10). The proposed fractional model is solved numerically using a generalized predictor-corrector of the Adams–Bashforth–Moulton method [33, 34]. The biological parameters estimated from the actual data reported in Pakistan from 1 March to 30 June 2020 and tabulated in Table 1 are utilized to obtain the simulation results. We varied different model parameters and the order *α* of the Caputo operator in order to explain the role of various parameters and memory index on the disease transmission patterns and control. The impact of contact rates $\zeta _{1},\zeta _{2}, \psi $ (a measure of social distancing effects), quarantine or contact-tracing policy $\varepsilon _{1}$, and hospitalization or self-isolation rate *τ* is depicted graphically in Figs. 2–6. The impact of environmental viral load due to the symptomatic $(m_{1} )$ and asymptomatic infected individuals $(m_{2} )$ is depicted in Figs. 7 and 8, respectively. Finally, the role of removal rate of virus from the environment (via disinfection spray etc.) is shown in Fig. 9.

### Effect of reduction in contact rates (a measure of social distancing)

First of all, we simulate COVID-19 model (10) for different values of effective contacts $\zeta _{1}$ in order to evaluate the impact of social distancing on the dynamics of cumulative symptomatic, asymptomatic, and hospitalized infected individuals. The simulation results for different four values of fractional order are shown in Fig. 2(a)–(d). It can be observed from these interpretations that the reduction in the effective contact rates $\zeta _{1}$ up to 50 percent to its baseline values reduces the peak of cumulative COVID-19 infected cases very well. The behavior is the same, seen for all four values of fractional order, although slightly faster decrease in pandemic peak is observed for smaller values of *α*. The impact of disease transmission rate due to the environmental viral load $\zeta _{2}$ is demonstrated in Fig. 3(a)–(d). With the reduction in this rate, a rapid decrease is observed in the total of symptomatic, asymptomatic, and hospitalized infected individuals and even becomes more biologically significant for smaller values of fractional order. This interpretation shows that the environmental transmission plays a significant role in disease prevalence. Finally, the influence of the reduction in *ψ* is depicted in Fig. 4(a)–(d) for four different values of *α*. A reasonable decrease in the pandemic peak is observed with a reduction of contact rate *ψ*. Overall, from these interpretations, we conclude that maintaining a strict social distancing and avoiding public gathering are needed to control the transmission of infection in the future.

### Effect of contact-tracing and hospitalization/self-isolation

The quarantine contact-tracing policy of exposed individuals and hospitalization/isolation of confirmed infected cases is another effective strategy to control the COVID-19 incidence. Here, we simulate the model with different levels of quarantine and hospitalization rates. The resulting graphical interpretation is shown in Figs. 5(a)–(d) and 6(a)–(d), respectively. A dramatic decrease in pandemic peak is observed with an increase (up to 50 percent) in the contact-tracing rate as shown in Fig. 5(a)–(d). The same behavior is obtained for all values of *α*. The influence of the increase in hospitalization of confirmed infected individuals is analyzed in Fig. 6(a)–(d). These results suggest that in the absence of treatment or vaccine, a proper and effective contact-tracing and self-isolation policy should be adopted until the elimination of pandemic.

### Effect of environmental viral load

The environmental viral load is one of the main routes of the COVID-19 infection transmission. In this section, we simulate model (10) with the reduction in the viral contribution to the environment by symptomatic and asymptomatic COVID-19 infective individuals at different rates. Figure 7(a)–(d) demonstrates the impact of the reduction in $m_{1}$ with different levels. It is observed that a reduction in $m_{1}$ (up to 50 percent) reduces the infected curve with a reasonable rate as seen in Fig. 7(a)–(d). The decrease in pandemic peaks is relatively more significant for smaller values of the fractional order of Caputo operator. The role of $m_{2}$ (viral contribution of asymptomatic individuals to the environment) is analyzed in Fig. 8(a)–(d). It can be seen that a reduction in $m_{3}$ at 50 percent to its baseline values given in Table 1 reduces the total number of symptomatic, asymptomatic, and hospitalized infected individuals very well. The similar behavior is obtained for all values of fractional order *α*. Finally, the influence of the removal rate of virus from the environment is depicted in Fig. 9(a)–(d). It is observed that enhancement in $m_{3}$ at 40 percent to its baseline value dramatically reduces the infection curves as shown in Fig. 9(a)–(d). This interpretation suggests that proper disinfection spray is necessary to remove the viruses from the environment in order to reduce the disease prevalence.

## Conclusion

The environmental viral load plays an essential role in the spread of the current COVID-19 pandemic. Limited research has been done on the environmental impacts on disease dynamics. In this paper, we studied the transmission dynamics of COVID-19 pandemic with quarantine, hospitalization (or self-isolation), and the environmental viral load impacts through a Caputo fractional model. Initially, the model is presented with classical integer order differential equations and then reformulated using fractional order operator with the power-law kernel. The basic mathematical analysis of the Caputo COVID-19 model is performed and the local and global stability of the disease-free case is proven. Utilizing the nonlinear least square approach, the model parameters are estimated and fitted for the COVID-19 actual cases recorded in Pakistan. The most important threshold quantity, known as the basic reproduction number, is presented both theoretically and numerically. The predictor-corrector iterative scheme of Adams–Moulton type is applied in order to solve the model numerically. The real estimated and fitted parameters are used to simulate the Caputo COVID-19 model. We depicted and discussed the simulation results for three different sets of model parameters showing the impact on the dynamics cumulative symptomatic, asymptomatic, and hospitalized infected cases. Firstly, we analyzed the influence of reduction in contact rates (a measure of social distancing) on the infected curves. The graphical interpretation of this case revealed that with a reduction of effective contacts with infected individuals, a significant decrease in the cumulative infected cases is seen. It is further observed that the reduction in the environmental viral load dramatically reduces the pandemic peak. Secondly, we explored the impact of the most commonly used interventions i.e., quarantine (or contact-tracing policy) and hospitalization (or self-isolation) on cumulative infected population. These graphical interpretations demonstrated that contact-tracing of exposed people is more effective in the elimination of infection. It can be seen that with an increase in the quarantine rate up to 50 percent to the base line value reduces the infected cases significantly. Finally, we have shown the impact of variation in the environmental viral load due to symptomatic and asymptomatic COVID-19 infected individuals. It is found that reducing the rate of viral release into the environment by asymptomatic infected individuals decreases the disease burden very well. It is because the asymptomatic infected individuals are unaware of the infection and freely continue the daily routine without any necessary safety precautions like the use of face mask and sanitizer. Moreover, we have also depicted the impact of variation in the removal rate of virus from the environment (or surfaces) on the disease prevalence. A dramatic reduction in the infected cases is found with an enhancement in this rate, which demonstrates that disinfection spray is very helpful in the disease elimination. We believe that the investigations in the paper will be beneficial for the health and decision-making authorities to combat the disease in the community. In the future, the present model can be reformulated by incorporating some suitable time-dependent control interventions using optimal control theory.

## Data Availability

Please contact the author for data requests.
